# Evaluating Curricular Impact on LGBTQ+ Health Competency: A Longitudinal Survey of Medical Students

**DOI:** 10.7759/cureus.105920

**Published:** 2026-03-26

**Authors:** Vicki Liu, Barbara Eckstein, Julie Armin

**Affiliations:** 1 Department of Family and Community Medicine, University of Arizona College of Medicine - Tucson, Tucson, USA; 2 Department of Family Medicine, Arizona Community Physicians, Tucson, USA

**Keywords:** health equity, health professions training, lgbtq+ health, medical education, sexual and gender minorities

## Abstract

Introduction

Lesbian, gay, bisexual, transgender, queer, and/or other sexual or gender minorities (LGBTQ+) populations experience disparities in healthcare access and outcomes due to discrimination and bias at individual, institutional, and societal levels. Although curricular interventions in medical education have shown promise, gaps remain in training, emphasizing a need for improved educational approaches to better prepare future clinicians. This study assesses the efficacy of two mandatory pre-clerkship sessions on increasing medical student awareness and shifting attitudes toward LGBTQ+ care.

Materials and methods

This study followed a single cohort of MD students at the University of Arizona College of Medicine - Tucson. Anonymous surveys containing 16 statements relating to sexual orientation and gender identity were administered at baseline, after a sexual orientation-focused (lesbian, gay, and bisexual (LGB)) session, and after a gender identity-focused (transgender and gender non-binary (TGNB)) session. Likert scale responses were collected to measure agreement and compare pre- and post-session results.

Results

The baseline, post-LGB session, and post-TGNB session surveys received 110, 89, and 56 responses, respectively. Across all 16 statements, both the percentage of students reporting agreement and the mean Likert scores increased after intervention, signifying improvement in self-reported awareness and attitudes toward LGBTQ+ care. At baseline, a particular area of weakness was student confidence in asking non-stigmatizing questions, with only 66% reporting comfort with discussing sexual orientation and 52% with discussing gender identity. Additionally, students reported lower awareness of issues pertaining to gender identity compared to sexual orientation. However, based on post-session results, curricular interventions effectively addressed these weaknesses.

Conclusion

These findings suggest that targeted educational sessions can positively shift medical students’ knowledge and sentiments toward LGBTQ+ care. Although this study was conducted within a single MD program, these results support the implementation of similar curricular interventions into other health professions training programs. By addressing gaps in confidence and knowledge regarding sexual orientation and gender identity, such efforts may arm future clinicians with the skills necessary to provide inclusive, affirming care.

## Introduction

Individuals who identify as lesbian, gay, bisexual, transgender, queer, and/or other sexual or gender minorities (LGBTQ+) are more likely to face discrimination in healthcare settings and experience poorer outcomes across various domains of physical and mental health [1-4]. These biases, whether explicit or implicit, exist at individual, institutional, and societal levels and arise from a complex interplay of social, cultural, and political factors [1]. Even within the larger LGBTQ+ community, healthcare is not experienced equally. For instance, transgender individuals report particularly high rates of delaying or avoiding medical care due to discrimination [4]. Moreover, intersectional identities across age, race and ethnicity, socioeconomic status, and other variables influence access to and quality of care among LGBTQ+ individuals [5-7].

In addition to systemic factors, providers play an important role in shaping an individual’s perception of healthcare. In a recent international study, LGBTQ+ patients often experienced implicit biases from providers in the form of misgendering and overlooking of sexual and gender diversity [8]. Thus, although reform on multiple fronts is needed, adequate training of future clinicians is one avenue to counteract disparities. Across numerous medical education programs, a wide variety of curricular interventions have been well-received by students and shown promise in enhancing student preparedness in serving LGBTQ+ populations [9-13]. Despite these initial strides, medical students continue to report inadequate guidance in these areas [14-16], particularly in relation to transgender and gender diverse topics [17-19]. Existing research has identified shortages in instruction time, standardized learning materials, LGBTQ+ examination content, trained faculty, and LGBTQ+-identifying role models [19]. Furthermore, one systematic review found that most studies examining curricular impact focused on knowledge and comfort levels, with few directly assessing implicit biases [20]. Based on these ongoing shortcomings in medical education, further optimization of educational interventions is necessary to equip future physicians with the knowledge and tools to provide equitable LGBTQ+ care.

This study closely follows medical students at a single institution to assess the effect of targeted educational sessions during the 18-month preclinical curriculum, with the specific objectives of evaluating changes in self-reported knowledge about LGBTQ+ healthcare, appraising shifts in self-reported attitudes toward LGBTQ+ populations, and identifying gaps to inform curricular improvements.

## Materials and methods

This study followed a single cohort of students in the MD program at the University of Arizona College of Medicine - Tucson. It was considered not human subjects research by the University of Arizona Institutional Review Board (STUDY00005064, notified August 7, 2024). As part of the pre-clerkship curriculum, students participated in two mandatory sessions, one in the first year of medical education that focuses on lesbian, gay, and bisexual (LGB) care and another in the second year centered around transgender and gender non-binary (TGNB) care. Each session was approximately 90 minutes and comprised a lecture-style presentation, small group conversations, and a panel discussion. Lecture content was developed by upperclassmen medical students who were leaders of the LGBTQ+ interest group, under the direct supervision of faculty with expertise in LGBTQ+ health. Small group discussions centered around case-based scenarios designed to encourage reflection and reinforce key concepts. Panel participants were LGB and TGNB community members who volunteered to provide personal insight, answer student questions, and share healthcare-related experiences. Session topics were selected based on national recommendations for LGBTQ+ health education and student feedback from previous years.

The primary outcomes were changes in self-reported awareness and attitudes toward LGBTQ+ care as the curriculum progressed. An anonymous Qualtrics survey was administered to the cohort at three timepoints: during medical school orientation, prior to any formal LGBTQ+ teaching (baseline); after the first session centered around sexual orientation (post-LGB session); and after the second session on gender identity (post-TGNB session). A total of 16 statements was curated to capture learning objectives and accepting attitudes pertaining to sexual orientation and gender identity. Survey items were curated by the study team and inspired by existing literature and guidelines surrounding LGBTQ+ health competencies. All statements were listed in the baseline survey. The post-LGB session survey only included the eight statements pertaining to sexual orientation, while the post-TGNB session assessed the other eight statements centered around gender identity. In each survey, students were prompted to rate their agreement with these statements based on a five-point Likert scale of strongly disagree (1), somewhat disagree (2), neither agree nor disagree (3), somewhat agree (4), or strongly agree (5). A higher Likert score, reflecting a greater extent of agreement, indicated curriculum effectiveness for the purposes of this study. To compare baseline versus post-session responses, the percentage of students answering somewhat or strongly agree at both timepoints was determined. The mean of baseline versus post-session Likert scale ratings was also calculated.

In addition, the initial baseline survey prompted responders to provide basic demographic information, including age and gender. Students’ familiarity with LGBTQ+ topics was also recorded, in the form of self-identification as part of the community, closeness to an LGBTQ+ individual, and prior LGBTQ+ education.

## Results

Of the 120 students in the class, 110 (91.7%) responded to the initial baseline survey. The median age of this group was 24 years. Sixty-four (58%) identified as women and 46 (42%) identified as men. Eighteen (16%) identified as part of the LGBTQ+ community, 85 (77%) did not, and the remaining 7 (6%) were unsure or preferred not to disclose. Seventy-eight (71%) indicated being close to someone who identifies as LGBTQ+, 25 (23%) did not, and 7 (6%) were unsure or preferred not to disclose. Sixty-seven (61%) reported receiving LGBTQ+-related education prior to medical school, 37 (34%) did not, and 6 (5%) were unsure or preferred not to disclose. 

The post-LGB session survey received 89 responses (74.2% response rate). Table 1 compares the extent of agreement students selected in response to statements pertaining to sexual orientation at the baseline versus post-LGB session timepoints.

**Table 1:** Likert scale responses to survey statements related to sexual orientation in the baseline survey (left columns) versus the post-LGB session survey (right columns). The percentage of students indicating some level of agreement and the mean Likert scale score are reported for each statement at both timepoints. LGB: lesbian, gay, and bisexual

Statement	Baseline survey (n = 110/120, 91.7% response rate)		Post-LGB survey (n = 89/120, 74.2% response rate)	
	% of students who somewhat or strongly agreed	Mean Likert scale score	% of students who somewhat or strongly agreed	Mean Likert scale score
I am aware of the unique health risks and barriers faced by lesbian, gay, and bisexual individuals.	78% (n = 86)	3.9	93% (n = 83)	4.4
I feel confident in my ability to establish rapport with lesbian, gay, and bisexual patients.	82% (n = 90)	4.1	93% (n = 83)	4.4
Lesbian, gay, and bisexual individuals deserve the same quality of medical care as heterosexual individuals.	97% (n = 107)	4.9	99% (n = 88)	4.9
When caring for lesbian, gay, and bisexual patients, I know how to ask non-stigmatizing questions about sexual orientation.	66% (n = 73)	3.7	94% (n = 84)	4.4
When caring for lesbian, gay, and bisexual patients, I know how to recognize implicit biases that I may have.	84% (n = 92)	4.1	93% (n = 83)	4.5
When caring for lesbian, gay, and bisexual patients, I know how to counteract implicit biases that I may have.	80% (n = 88)	4.0	91% (n = 81)	4.3
When caring for lesbian, gay, and bisexual patients, I know how to speak up against discrimination and/or microaggressions from others.	81% (n = 89)	4.2	89% (n = 79)	4.4
When caring for lesbian, gay, and bisexual patients, I know how to advocate for inclusive approaches to discuss sexual orientation.	74% (n = 81)	4.1	90% (n = 80)	4.4

The post-TGNB session survey received 56 responses (46.7% response rate). Table 2 compares Likert scale responses surrounding statements about gender identity from the baseline versus post-TGNB session surveys.

**Table 2:** Likert scale responses to survey statements related to gender identity in the baseline survey (left columns) versus the post-TGNB session survey (right columns). The percentage of students indicating some level of agreement and the mean Likert scale score are reported for each statement at both timepoints. TGNB: transgender and gender non-binary

Statement	Baseline survey (n = 110/120, 91.7% response rate)		Post-TGNB survey (n = 56/120, 46.7% response rate)	
	% of students who somewhat or strongly agreed	Mean Likert scale score	% of students who somewhat or strongly agreed	Mean Likert scale score
I am aware of the unique health risks and barriers faced by transgender and non-binary individuals.	71% (n = 78)	3.8	96% (n = 54)	4.6
I feel confident in my ability to establish rapport with transgender and non-binary patients.	74% (n = 81)	3.9	93% (n = 52)	4.4
Transgender and non-binary individuals deserve the same quality of medical care as cisgender individuals.	99% (n = 109)	4.9	100% (n = 56)	4.9
When caring for transgender and non-binary patients, I know how to ask non-stigmatizing questions about gender identity.	52% (n = 57)	3.5	95% (n = 53)	4.5
When caring for transgender and non-binary patients, I know how to recognize implicit biases that I may have.	80% (n = 88)	4.0	95% (n = 53)	4.6
When caring for transgender and non-binary patients, I know how to counteract implicit biases that I may have.	76% (n = 84)	3.9	91% (n = 51)	4.5
When caring for transgender and non-binary patients, I know how to speak up against discrimination and/or microaggressions from others.	80% (n = 88)	4.2	89% (n = 50)	4.5
When caring for transgender and non-binary patients, I know how to advocate for inclusive approaches to discuss gender identity.	77% (n = 85)	4.2	95% (n = 53)	4.6

## Discussion

This study substantiates that targeted educational sessions can effectively develop medical students’ knowledge and sentiments pertaining to LGBTQ+ populations and healthcare. Across all evaluated areas, post-session results showed demonstrable improvement, with increases in both the proportion of students reporting agreement and the extent of overall agreement based on Likert scores. At baseline, comfort in asking non-stigmatizing questions and knowledge of gender identity-related issues were particularly low. However, these initial gaps were effectively addressed by the sexual orientation- and gender identity-focused sessions, respectively.

These findings largely align with previous studies demonstrating the value of structured LGBTQ+ education [9-13]. Given the ongoing disparities faced by LGBTQ+ individuals [1-7], optimizing provider competence through early training represents a practical step toward more equitable healthcare. Rather than traditional diversity training, explicitly focusing on LGBTQ+ topics, particularly with thoughtful integration of lived experiences [21,22], enables deeper exploration of the nuances involved in caring for this community. Incorporating such content into medical training in a methodical, intentional manner carries the potential to reduce implicit and explicit biases in patient interactions. Furthermore, creating space for scholarly exposure to LGBTQ+ health topics may contribute to shaping professional norms around inclusive, non-stigmatizing inquiry and culturally sensitive care.

This study has several limitations. As response rates declined from baseline to post-session surveys, selection bias and reliance on self-reported measures may misconstrue results. Due to a lack of time, the post-TGNB survey was sent out via an email listserv as opposed to asking students to complete the survey in-person at the end of the session, which likely resulted in the substantial decline in response rate. Additionally, evaluating a single cohort at one institution limits the overall generalizability of the findings. Notably, a considerable proportion of students in this study personally identified as LGBTQ+ (16%), were close to LGBTQ+ individuals (71%), and/or had prior education on LGBTQ+ topics (61%). Students’ baseline attitudes and exposure to LGBTQ+ populations likely have a strong influence on the effectiveness of curricular interventions. In the present cohort, this made it difficult to fully examine changes in student attitudes, as baseline sentiments were already highly supportive of LGBTQ+ individuals. Thus, additional research should be done in various educational settings with diverse student bodies. Future research should also incorporate qualitative student feedback and examine longitudinal effects of these interventions, including their impact on clinical interactions once students advance beyond pre-clerkships. Developing a standardized methodology to assess LGBTQ+ health competencies and educational gaps would be highly beneficial [23-25] and enable potential replication across academic settings. Looking forward, evaluating strategies for sustained integration of LGBTQ+ health topics into medical curricula, along with stronger objective measures of effectiveness, would be critical to advancing equitable care for this population.

## Conclusions

Altogether, this longitudinal survey of medical students at a single institution demonstrates that brief, targeted educational sessions can meaningfully enhance self-reported awareness, attitudes, and confidence in providing care for LGBTQ+ populations. Gaps at baseline, particularly surrounding gender identity-related topics and non-stigmatizing discussion of LGBTQ+ identity, were effectively addressed through these sessions. These positive outcomes indicate that similar curricular interventions could be successfully adapted and implemented at other medical schools and health professions programs. Incorporating structured education on LGBTQ+ health early in training not only strengthens knowledge and skills but also fosters more inclusive and affirming mindsets and practices. These results underscore the value of intentional, evidence-based curricular strategies in preparing future clinicians to meet diverse patient needs and potentially alleviate health disparities.
